# Dietary phytochemical index is favorably associated with oxidative stress status and cardiovascular risk factors in adults with obesity

**DOI:** 10.1038/s41598-023-34064-4

**Published:** 2023-04-29

**Authors:** Soudabeh Hamedi-Shahraki, Mohammad-Reza Jowshan, Mohammad-Amin Zolghadrpour, Farshad Amirkhizi, Somayyeh Asghari

**Affiliations:** 1 Department of Epidemiology and Biostatistics, Faculty of Public Health, Zabol University of Medical Sciences, Zabol, Iran; 2 Department of Clinical Nutrition, School of Nutritional Sciences and Dietetics, Tehran University of Medical Sciences, Hojjatdoust St., Naderi St.,, No#44, Keshavarz Blvd, 141556117 Tehran, Iran; 3 Department of Nutrition, Faculty of Public Health, Zabol University of Medical Sciences, Zabol, Iran

**Keywords:** Obesity, Nutrition

## Abstract

Phytochemicals are bioactive compounds found in plant-based foods. Consumption of phytochemical-rich foods has been associated with cardiovascular and metabolic diseases prevention in various populations. To quantify the phytochemical content of the diet, dietary phytochemical index (DPI) was established which is defined as the proportion of daily energy intake derived from foods rich in phytochemicals. The purpose of this study was to evaluate the association between the DPI and oxidative stress markers and cardiovascular risk factors in obese adults. In this cross-sectional study, a total of 140 adults aged 20–60 years and body mass index (BMI) of ≥ 30 kg/m^2^ were included. A validated food frequency questionnaire (FFQ) was used to collect information on dietary intakes. The DPI was calculated based on the following formula: DPI = [daily energy obtained from foods rich in phytochemicals (kcal)/total daily energy intake (kcal) × 100]. There was an inverse association between DPI and serum concentrations of Malondialdehyde (MDA) (*P* = 0.004), triglyceride (TG) (*P*-trend = 0.003), high-sensitive C-reactive protein (hs-CRP) (*P* = 0.017), and erythrocyte superoxide dismutase (SOD) activity (*P* = 0.024). Total antioxidant capacity (TAC) was positively associated with DPI score (*P* = 0.045). No significant relationship was found between the DPI score and fasting blood sugar (FBS), total cholesterol (TC), high-density lipoprotein cholesterol (HDL-C), low-density lipoprotein cholesterol (LDL-C), total oxidant status (TOS), glutathione peroxidase (GPx), catalase (CAT), and anthropometric parameters as well as systolic and diastolic blood pressure. The current study found that there was a significant inverse association between DPI and oxidative stress, inflammation, and hypertriglyceridemia as cardiovascular disease (CVD) risk factors in obese population. However, further research is needed to confirm these findings.

## Introduction

Cardiovascular disease (CVD) is the major cause of morbidity and death worldwide^1^. According to the recent studies, it has been shown that the disease has expanded to younger age groups as well^2^. This is partly attributed to the global epidemic of obesity, which has been demonstrated to be one of the most important predictors of CVD. Several risk factors related to CVD, such as hypertension, dyslipidemia, and insulin resistance are exacerbated by obesity^3^.

Oxidative stress and inflammation have been described in obesity and the pathogenesis of these CVD risk factors^4^. Oxidative stress is a state caused by the overproduction of reactive species, known as pro-oxidants, and the incapability of the antioxidant defense system to scavenge these species, which consequently causes cellular damage and destruction^5^. Several observational studies have supported the association between oxidative imbalance and cardio-metabolic conditions, which is associated with an increased risk of CVD. Thus, oxidative stress could be a potential target to manage cardiovascular complications in obese subjects.

Diet is considered as a key modifiable component of lifestyle in primary prevention of CVD and its consequences. Studies have shown that adherence to the plant-based diets and higher consumption of fruits and vegetables, whole grains, and dietary fiber may reduce the risk of developing CVD^6,7^. It is assumed that the protective effects of the plant-based diets may be attributed to dietary phytochemicals, in part^8^. Phytochemicals are natural bioactive compounds found in plants such as fresh fruits, vegetables, nuts, whole grains, and legumes^9^ with various known health benefits such as anti-inflammatory, antioxidant, antiangiogenic, and antihypercholesterolemic effects^10^.

Previous epidemiologic studies have demonstrated that the consumption of phytochemical-rich foods can help prevent cardiovascular and metabolic diseases in various populations. Evidence also suggest that phytochemicals can reduce the levels of inflammatory and oxidative stress markers and improve serum glycemic and lipid profiles^11^.

To quantify the phytochemical content of the diet, McCarty et al. established a simple and practical tool, known as dietary phytochemical index (DPI) which is defined as the proportion of daily energy intake derived from foods rich in phytochemicals^12^. Diet quality may be determined from this simple method for assessing phytochemical consumption^13^. It has been shown that higher DPI scores are associated with a lower risk of chronic diseases such as obesity, diabetes, metabolic syndrome, cancer, and CVD^14^. The available data regarding the association between DPI and oxidative stress biomarkers as well as other cardiometabolic risk factors in obese individuals are limited and the existing data are highly controversial. Thus, the purpose of this study was to investigate the relationship between DPI and oxidative stress status and cardiovascular risk factors in adults with obesity.

## Materials and methods

### Study subjects

This cross-sectional study was conducted on 140 obese male and female subjects with a body mass index (BMI) of ≥ 30 kg/m^2^ and the age range of 20–60 years who attended the out-patient clinics affiliated to Zabol University of Medical Sciences. The sample size was determined based on the information obtained from the study by Golzarand et al.^15^ for serum triglyceride (TG) concentrations as the dependent variable employing the formula N = [(Z_1-α/2_)^2^S^2^]/d^2^. Considering SD = 7.0, d = 1.2, and α = 0.05, it was calculated that 130 subjects needed to be selected for the study. The sample size of the study was increased to 140 subjects for a possible dropout of 10%.

Pregnant and lactating women; those with recent inflammatory and infectious conditions, surgery or major trauma, a prior history of stroke, and renal or hepatic diseases; as well as those who were taking any antioxidant supplements three months before the study enrollment were not included. Subjects who were on a specific diet and subjects with obesity due to genetic causes or endocrine disorders were also not included.

### Ethics approval and consent to participate

Prior to the study enrollment, participants were fully informed about the aims and the protocol of the study. Then, subjects were asked to sign a written informed consent. The study was performed in accordance with the Declaration of Helsinki and the protocol of the research was approved by the Ethics Committee of Zabol University of Medical Sciences (Ethics No: IR.ZBMU.REC.1400.118).

### Demographic factors and anthropometric assessments

All participants enrolled in the study were interviewed by trained personnel. An interviewer-administered questionnaire was applied to gather the relevant demographic characteristics, detailed clinical information, and lifestyle habits of participants.

Weight was measured using a digital scale in the standing position with sensitivity of 100 g while subjects were light clothing, without shoes. Height was measured to the nearest of 0.5 cm in the normal standing position without shoes using a fixed wall scale. BMI was calculated as body weight (kg) divided by height in meters squared (m^2^). Waist circumference (WC) was measured using a flexible tape measure, without imposing any pressure to body surface.

### Physical activity, and blood pressure assessment

The subject’s physical activity was assessed through the short form of the International Physical Activity Questionnaire (IPAQ)^16^, then divided into three categories of *“high”*, *“moderate”*, and *“heavy”* activity.

Blood pressure was measured after a 15-min sitting in a quiet environment using a mercury sphygmomanometer. The mean systolic blood pressure (SBP) and diastolic blood pressure (DBP) were recorded using two readings measured at 5-min intervals.

### Dietary assessment and DPI calculation

Dietary intakes were evaluated using a validated semi-quantitative food frequency questionnaire (FFQ) with 168 food items^17^. A trained nutritionist recorded the subject’s frequency of food consumption in the previous year on a daily (e.g. bread), weekly (e.g. rice, meat) or monthly (e.g. fish) basis through face-to-face interviews. The assistants helped subjects estimate food quantities using calibrated household measurements (e.g. spoon, bowl, ladles). Portion sizes were then converted to grams. The intake of calorie and nutrients were calculated using Nutritionist IV software (First Databank; Hearst, San Bruno, CA, USA) based on the Iranian foods-modified US Department of Agriculture food composition. Nearly all foods in the participant list were coded and non-available foods were coded to a similar item.

The DPI was calculated based on the method developed by McCarty^12^ on the following formula: DPI = [daily energy obtained from foods rich in phytochemicals (kcal)/total daily energy intake (kcal)) × 100]. Fruits, vegetables, legumes, whole grains, nuts, soy products, seeds, and olive oil were considered as the phytochemical-rich foods to calculate DPI. Potatoes and pickled and powdered vegetables were not considered in the calculations because of their low phytochemical content. Furthermore, natural fruit juices were classified into the fruit group and vegetable juices and tomato sauces were classified into the vegetable group due to their high phytochemical content; therefore, they were included in the calculation of DPI. After calculating the DPI scores, they were categorized into tertiles, while subjects in the top tertile have the highest score of DPI.

### Biochemical measurements

Fasting blood samples were taken after 10–12 h from all study subjects and centrifuged at 3500 rpm (~ 2000 g) to separate the sera. Fasting blood sugar (FBS), total cholesterol (TC), high-density lipoprotein cholesterol (HDL-C), low-density lipoprotein cholesterol (LDL-C), and TG concentrations were assessed enzymatically on the day of sampling by commercial kits (Pars-Azmoon Co., Tehran, Iran) and the remaining sera were stored at − 80 °C until the assays were performed. Serum concentrations of high-sensitive C-reactive protein (hs-CRP) were measured based on the immunoturbidimetric method using the commercial kit (Pars Azmoon Co., Tehran, Iran) which is sensitive to 0.1 mg/L variations in serum concentrations of hs-CRP. The erythrocyte superoxide dismutase (SOD, EC 1.15.1.1) and glutathione peroxidase (GPx, EC 1.11.1.9) activities were determined using the Ransod (Randox Laboratories, Ltd., UK, cat. no. SD-125) and Ransel (Randox Laboratories, Ltd., UK, cat.no. RS-504) kits, respectively. Erythrocytes catalase (CAT, EC 1.11.1.6) activity was evaluated based on the method developed by Hygo Aebi^18^ by following the decomposition of H_2_O_2_ in phosphate buffer of pH 7.2 spectrophotometrically at 230 nm.

Serum concentrations of total antioxidant capacity (TAC) were determined colorimetrically in triplicate samples using 2, 2'-Azino-di-[3-ethylbenzthiazoline sulphonate] (ABTS)^19^. Malondialdehyde (MDA) concentrations in serum were also assessed by the measurement of thiobarbituric acid reactive substances (TBARS) according to Uchihara and Mihara method^20^. Serum total oxidant status (TOS) was determined according to Erel method^21^. The basis of this method depends on the oxidation of Fe^2+^ to Fe^3+^ in the presence of the oxidants contained in the sample. Fe^+3^ forms a colored complex with xylenol orange which the color intensity varies according to the amount of oxidant in the sample. The absorbance of this color spectrophotometrically is read at 530 nm. The assay is calibrated with hydrogen peroxide (H_2_O_2_) and the results are expressed in terms of micromolar H_2_O_2_ equivalent per liter (μmol H_2_O_2_ Equiv./L).

### CVD risk factors

In this study, hyperglycemia and dyslipidemia were defined based on the diagnostic criteria proposed by the Adult Treatment Panel III guidelines of the National Cholesterol Education Program (NCEP ATP III)^22^. Hyperglycemia was considered as FBS ≥ 5.55 mmol/L or currently taking medication for impaired fasting glucose. Hypercholesterolemia was defined as TC ≥ 6.22 mmol/L or being on the treatment with lipid-lowering agents. Subjects who had serum TG ≥ 1.69 mmol/L or were on triglyceride-lowering medications were diagnosed with hypertriglyceridemia. Furthermore, “high LDL-C” and “low HDL-C” among subjects were diagnosed if they had serum LDL-C concentration of > 4.14 mmol/L and serum HDL-C concentration < 1.04 mmol/L, respectively. Based on the Eighth Joint National Committee on Prevention, Detection, Evaluation and Treatment of High Blood Pressure^23^, subjects who had SBP ≥ 140 mmHg or DBP ≥ 90 mmHg or were on treatment with antihypertensive drugs were diagnosed with hypertension. According to the cutoff proposed by the American Heart Association and Centers for Disease Control, serum hs-CRP concentrations of ≥ 3.0 mg/l were considered as “high hs-CRP^24^”.

### Statistical analyses

Statistical analyses were done using SPSS software (version 25; SPSS, Chicago, IL, USA). Data are illustrated as mean ± standard deviation (SD) for normally distributed continuous variables and median (interquartile range) for non-normally distributed variables. Categorical variables are displayed as absolute numbers and frequencies (%). The normality of data distribution was appraised using the Kolmogorov–Smirnov test. Subjects were classified based on cut-points of DPI in tertiles categories as follows: first tertile, < 27.3; second tertile, 27.3 to 33.9; third tertile, > 33.9. Differences in the characteristics of subjects across tertiles of DPI were evaluated by one-way analysis of variance (ANOVA) or chi-squared test, as appropriate. Sex-, age-, and energy-adjusted means for dietary intakes across DPI categories were compared by analysis of covariance (ANCOVA). Comparison of metabolic, inflammatory, and oxidative stress markers across DPI categories were also performed using ANOVA test for normally distributed data. To investigate and find the actual significant group differences, ANOVA was followed by Tukey’s test. Non-normally distributed data were compared by Kruskal–Wallis test across DPI categories and followed by Dunn’s post hoc test to investigate the actual significant group differences. The Jonckheere-Terpstra test was used to determine whether there is a significant trend between DPI and non-normally distributed variables. To find the association between DPI and metabolic, inflammatory, and oxidative stress biomarkers, the linear regression analysis was applied.

Logistic regression was used to investigate the relationship of DPI and CVD risk factors in two different models: the adjusted and unadjusted models. In the adjusted model, sex (male, female), age (years), BMI (kg/m^2^), smoking (yes, no), and physical activity level (categorical: light, moderate, and heavy) were adjusted. In both models, the first tertile of DPI was considered as the reference category. In all analyses, *P* < 0.05 was considered statistically significant.

## Results

### Subject’s characteristics

In this study, 140 obese subjects were included in the study. The mean age of participants was 41.0 ± 7.3 years and 55.0% of them were males. The DPI in this study ranged from 14.9 to 56.0 with the mean ± SD of 30.7 ± 7.1. The DPI in the first, second, and third tertiles was < 27.3, 27.3 to 33.9 and > 33.9, respectively. General characteristics of participants across tertile categories of DPI are shown in Table 1. There were no significant differences in the mean age, SBP, and DBP values as well as the distribution of participants regarding smoking, physical activity status, and education level among study groups.

**Table 1:** Characteristics of subjects with obesity across tertiles of DPI. Values are expressed as mean ± standard deviation unless indicated otherwise. DPI dietary phytochemical index, BMI body mass index, WC waist circumference, SBP systolic blood pressure, DBP diastolic blood pressure. ^†^Tertile cut-points of DPI are as follows: first tertile, < 27.3; second tertile, 27.3 to 33.9; third tertile, > 33.9. ^**‡**^Obtained from one-way ANOVA test or Pearson chi-square test for continuous or categorical variables, respectively.

Risk factor	Tertiles of DPI^†^			*P* ^**‡**^
	T1 (n = 47)	T2 (n = 47)	T3 (n = 46)	
Male, n (%)	27 (57.4)	28 (59.6)	22 (47.8)	0.480
Age (years)	42.0 ± 7.0	39.8 ± 7.8	41.2 ± 7.1	0.618
SBP (mmHg)	124.1 ± 20.8	120.2 ± 12.4	123.5 ± 18.5	0.870
DBP (mmHg)	78.7 ± 9.2	75.6 ± 13.2	75.8 ± 9.3	0.187
Smoking, n (%)	16 (34.0)	13 (27.7)	13 (28.3)	0.758
Physical activity, n (%)				
Light	36 (76.6)	40 (85.1)	37 (80.40)	0.568
Moderate	8 (17.0)	4 (8.5)	4 (8.7)	
Heavy	3 (6.4)	3 (6.4)	5 (10.9)	
Education level				
Primary school	11 (23.4)	13 (27.7)	9 (19.6)	0.899
Secondary & high school	23 (48.9)	20 (42.5)	22 (47.8)	
Diploma & university	13 (27.7)	14 (29.8)	15 (32.6)	

### Dietary intakes

Daily dietary intakes of study subjects across tertiles of DPI are presented in Table 2. Subjects in the upper tertile categories of DPI had higher energy intakes (*P* = 0.001) and higher daily energy intakes from carbohydrates (*P* = 0.004). Those in the highest tertile categories of DPI had higher intakes of fiber (*P* < 0.0001), vitamin C (*P* = 0.009), and folate (*P* = 0.006) compared with those in the lower tertiles. Compared to the subjects in the lowest tertile, those in the highest tertile of DPI consumed more grains (*P* < 0.0001), fruits (*P* < 0.0001), vegetables (*P* < 0.0001), and olive sources (*P* < 0.0001). No significant differences were found in daily energy intakes from protein, total fat, saturated fatty acids (SFA), monounsaturated fatty acids (MUFA), and polyunsaturated fatty acids (PUFA) across tertile categories of DPI. In addition, there were no significant differences across tertile categories of DPI for intakes of vitamin A, vitamin E, calcium, zinc, meats, nuts, and dairy products.

**Table 2:** Dietary intakes of subjects with obesity across tertiles of DPI. Data are expressed as mean ± standard deviation. DPI dietary phytochemical index, RAE retinol activity equivalents.

Variables	Tertiles of DPI^†^			*P*^**‡**^
	T1 (n = 47)	T2 (n = 47)	T3 (n = 46)	
Energy intake (kcal)^**§**^	2239 ± 76	2407 ± 83*^,^^#^	2674 ± 89*^,^^#,##^	0.001
Nutrients				
Carbohydrate (% of energy)	51.2 ± 2.9	55.9 ± 3.8	62.3 ± 4.8*^,^^#^	0.004
Protein (% of energy)	12.4 ± 2.7	13.7 ± 2.0	12.6 ± 3.1	0.219
Total fat (% of energy)	38.3 ± 6.6	37.2 ± 6.1	34.7 ± 7.4	0.087
SFA (% of energy)	8.6 ± 2.8	10.1 ± 3.7	9.8 ± 2.4	0.286
MUFA (% of energy)	7.5 ± 3.1	8.4 ± 2.8	7.7 ± 2.9	0.345
PUFA (% of energy)	5.4 ± 2.2	5.7 ± 2.6	6.0 ± 2.8	0.102
Total fiber (g/day)	20.7 ± 2.1	24.5 ± 2.8	30.6 ± 3.0*^,^^#^	< 0.001
Vitamin A (RAE/day)	697 ± 195	743 ± 271	756 ± 312	0.086
Vitamin C (mg/day)	126 ± 61	143 ± 58	157 ± 60*^,^^#^	0.009
Vitamin E (mg/day)	16.0 ± 8.3	15.8 ± 6.7	16.4 ± 7.2	0.417
Folate (mcg/day)	220 ± 79	290 ± 84*^,^^#^	308 ± 85*^,^^#^	0.006
Calcium (mg/day)	913 ± 204	906 ± 199	945 ± 228	0.320
Zinc (mg/day)	11.2 ± 5.8	11.8 ± 5.1	10.4 ± 4.7	0.401
Food groups				
Meats (g/day)	89.3 ± 44.1	96.5 ± 48.2	92.7 ± 50.7	0.205
Grains (g/day)	319 ± 108	370 ± 125*^,^^#^	431 ± 133*^,^^#^	0.002
Fruits (g/day)	185 ± 74	227 ± 98	307 ± 94*^,^^#,##^	< 0.001
Vegetables (g/day)	197 ± 87	285 ± 94*^,^^#^	298 ± 80*^,^^#^	< 0.001
Nuts (g/day)	5.0 ± 1.9	5.5 ± 2.3	4.1 ± 1.5	0.407
Legumes (g/day)	15.8 ± 5.2	16.1 ± 6.4	14.9 ± 5.3	0.097
Dairy products (g/day)	192 ± 61	190 ± 49	186 ± 57	0.231
Olive and olive oil (g/day)	1.24 ± 0.50	2.06 ± 0.71*^,^^#^	2.53 ± 0.65*^,^^#^	< 0.001

^†^Tertile cut-points of DPI are as follows: first tertile, < 27.3; second tertile, 27.3 to 33.9; third tertile, > 33.9.

^**‡**^Obtained from analysis of covariance (ANCOVA).

^**§**^Energy intake is adjusted for sex and age; all other values are adjusted for age, sex and energy intake.

*P < 0.05 (Obtained from Tukey post hoc test).

^#^Significant difference from first tertile.

^##^Significant difference from second tertile.

### Association of DPI with metabolic, inflammatory, oxidative stress markers, and CVD risk factors

Table 3 indicates the anthropometric indices and metabolic, inflammatory, and oxidative stress markers of study subjects across tertiles of DPI. There were no significant differences in the mean weight, BMI, and WC across tertiles of DPI. The mean serum concentrations of TG (*P*-trend = 0.005) and hs-CRP (*P*-trend = 0.002) had decreasing trends across the increasing in DPI scores. The subjects in third tertile of DPI had lower serum concentrations of TG and hs-CRP compared to the counterparts in the first tertile (*P* < 0.05 for both). No significant regular trend was observed regarding FBS, TC, LDL-C, and HDL-C across DPI tertile categories. There was a trend towards decreasing serum concentrations of MDA (*P*-trend = 0.001) and TOS (*P*-trend = 0.013) as well as erythrocytes SOD activity (*P*-trend = 0.013) with increasing tertiles of DPI. Indeed, the subjects in the top tertile of DPI had lower serum concentrations of MDA and TOS as well as erythrocytes SOD activity compared to the subjects in the first tertile (*P* < 0.05 for all). Inversely, serum TAC concentration had increasing trend across the increasing in DPI categories (*P*-trend = 0.015) so that, subjects in the third tertile of DPI had higher serum TAC concentration compared to the counterparts in the first tertile (*P* < 0.05). Nonetheless, no significant associations was observed between DPI with GPx and CAT activities (Table 3).

**Table 3:** Anthropometric indices and metabolic, inflammatory, and oxidative stress markers of subjects with obesity across tertiles of DPI.

Risk factor	Tertiles of DPI^†^			*P***-**trend^**‡**^
	T1 (n = 47)	T2 (n = 47)	T3 (n = 46)	
Weight (kg)	96.7 ± 10.8	96.2 ± 12.9	92.3 ± 14.3	0.092
BMI (kg/m^2^)	32.3 ± 2.7	31.8 ± 2.8	32.3 ± 2.7	0.505
WC (cm)	106.9 ± 10.2	104.8 ± 9.1	103.8 ± 9.4	0.129
TC (mmol/L)	5.21 ± 0.91	5.14 ± 0.88	4.94 ± 0.84	0.128
TG (mmol/L)	2.08 ± 0.35	1.91 ± 0.37	1.88 ± 0.29	0.005
LDL-C (mmol/L)	3.63 ± 0.77	3.42 ± 0.69	3.42 ± 0.74	0.172
HDL-C (mmol/L)	1.31 ± 0.27	1.2 ± 0.22	1.23 ± 0.28	0.137
FBS (mmol/L)	5.64 ± 1.24	5.74 ± 1.04	5.33 ± 0.83	0.166
hs-CRP (mg/L)	7.20 (4.12, 9.34)	6.10 (2.96, 8.17)	3.99 (2.41, 8.17)*^,^^#^	0.002
MDA (nmol/mL)	1.90 (1.77, 2.10)	1.80 (1.59, 2.0)	1.71 (1.57, 1.85)*^,^^#^	0.001
TOS (µmol H_2_O_2_ Equiv./L)	14.0 (9.4, 19.2)	9.9 (8.2, 12.5)*^,^^#^	9.6 (8.2, 12.8)*^,^^#^	0.013
TAC (mmol/L)	1.64 ± 0.48	1.76 ± 0.37	1.92 ± 0.42**^,^^#^	0.015
SOD (U/gHb)	1182 ± 174	1098 ± 177**^,^^#^	1082 ± 167**^,^^#^	0.013
GPx (U/gHb)	42.4 (32.2, 52.7)	44.9 (37.3, 59.1)	43.8 (32.7, 58.5)	0.246
CAT (K/gHb)	211 (192, 245)	216 (200, 291)	224 (194, 258)	0.273

*DPI* dietary phytochemical index, *TC* total cholesterol, *TG* triglyceride, *LDL-C* low density lipoprotein cholesterol, *HDL-C* high density lipoprotein cholesterol, *FBS* fasting blood sugar, *MDA* malondialdehyde, *TOS* total oxidant status, *TAC* total antioxidant capacity, *SOD* superoxide dismutase, *GPx* glutathione peroxidase, *CAT* catalase, *hs-CRP* high-sensitive C-reactive protein.

Values are expressed as mean ± standard deviation for normally distributed data (i.e*.* TC, TG, LDL-C, HDL-C, FBS, TAC, and SOD) and median (interquartile range) are presented for data not normally distributed (i.e*.*MDA, TOS, GPX, CAT, and hs-CRP).

^†^Tertile cut-points of DPI are as follows: first tertile, < 27.3; second tertile, 27.3 to 33.9; third tertile, > 33.9.

^‡^Obtained from analysis of variance (ANOVA) for normally distributed data (i.e. TC, TG, LDL-C, HDL-C, FBS, TAC, and SOD) and the non-parametric Kruskale-Wallis test for not normally distributed data (i.e. MDA, TOS, GPX, CAT, and hs-CRP).

*P < 0.05 (Obtained from Dunn’s post hoc test).

**P < 0.05 (Obtained from Tukey post hoc test).

^#^Significant difference from first tertile.

The results obtained from the multiple linear regression analysis confirms aforementioned relationships (Table 4). DPI was inversely associated with serum concentrations of MDA (*β* =  − 0.244, *P* = 0.004), TG (*β* =  − 0.253, *P* = 0.003), and hs-CRP (*β* =  − 0.202, *P* = 0.017) as well as erythrocytes SOD activity (*β* =  − 0.189, *P* = 0.024) after controlling for potential confounders. In addition, DPI was positively associated with serum concentrations of TAC (*β* = 0.171, *P* = 0.045).

**Table 4:** Results of multiple linear regression analysis that evaluated the association between dependent (metabolic, inflammatory, and oxidative stress markers) and independent variables (DPI) (*n* = 140).

Variables	DPI score		
	B (S.E.)	β	*P*^†^
MDA (nmol/mL)	− 0.012 (0.004)	− 0.244	0.004
TOS (µmol H_2_O_2_ Equiv./L)	− 0.097 (0.067)	− 0.122	0.148
TAC (mmol/L)	0.010 (0.005)	0.171	0.045
SOD (U/gHb)	− 4.746 (2.077)	− 0.189	0.024
GPx (U/gHb)	0.144 (0.182)	0.065	0.431
CAT (K/gHb)	− 0.020 (0.803)	− 0.002	0.980
TC (mg/dL)	− 0.966 (0.396)	− 0.034	0.216
TG (mg/dL)	− 1.097 (0.362)	− 0.253	0.003
LDL-C (mg/dL)	− 0.747 (0.338)	− 0.088	0.129
HDL-C (mg/dL)	− 0.086 (0.122)	− 0.061	0.481
FBS (mg/dL)	− 0.126 (0.227)	− 0.047	0.580
hs-CRP (mg/L)	− 0.087 (0.036)	− 0.202	0.017

*DPI* dietary phytochemical index, *MDA* malondialdehyde, *TOS* total oxidant status, *TAC* total antioxidant capacity, *SOD* superoxide dismutase, *GPx* glutathione peroxidase, *CAT* catalase, *TC* total cholesterol, *TG* triglyceride, *LDL-C* low density lipoprotein cholesterol, *HDL-C* high density lipoprotein cholesterol, *FBS* fasting blood sugar, *hs-CRP* high-sensitive C-reactive protein, *B* unstandardized coefficient, *S.E.* standard error.

^†^Adjusted for age, sex, BMI, cigarette smoking, and physical activity.

The odds ratios (OR) and 95% confidence interval (CI) for the incidence of CVD risk factors as the dependent variables across tertile categories of DPI as independent variables are illustrated in Table 5. After adjustment for potential confounding factors including age, sex, BMI, cigarette smoking, and physical activity, a noticeable inverse trend was found between DPI and the odds of having the hypertriglyceridemia (OR among tertiles, T1 to T3: 1.00, 0.58, and 0.32, respectively; *P*-trend = 0.023) and high hs-CRP (OR among tertiles, T1 to T3: 1.00, 0.49, and 0.23, respectively; *P*-trend = 0.004). However, no significant associations was found between DPI and the probability of having the hypercholesterolemia, high LDL-C, low HDL-C, hyperglycemia, and hypertension.

**Table 5:** Crude and adjusted OR (95%CI) for incidence of cardiovascular risk factors in subjects with obesity across tertiles (T) of DPI.

DPI and cardiometabolic risk factors	Simple logistic regression	Multiple logistic regression
	Crude OR (95.0% C.I.)	Adj. OR (95.0% C.I.)^**‡**^
Hypercholesterolemia		
T1^**§**^ (Reference)	1.0	1.0
T2	0.60 (0.21, 1.46)	0.55 (0.20, 1.46)
T3	0.50 (0.18, 1.33)	0.47 (0.17, 1.29)
*P*-trend^†^	0.150	0.143
Hypertriglyceridemia		
T1^**§**^ (Reference)	1.0	1.0
T2	0.61 (0.26, 1.47)	0.58 (0.24, 1.43)
T3	0.34 (0.13, 0.89)	0.32 (0.12, 0.86)
*P*-trend^†^	0.025	0.023
High LDL-C		
T1^**§**^ (Reference)	1.0	1.0
T2	0.66 (0.27, 1.62)	0.71 (0.29, 1.77)
T3	0.46 (0.18, 1.21)	0.47 (0.18, 1.22)
*P*-trend^†^	0.114	0.120
Low HDL-C		
T1^**§**^ (Reference)	1.0	1.0
T2	0.55 (0.24, 1.24)	0.52 (0.22, 1.21)
T3	0.52 (0.22, 1.18)	0.49 (0.21, 1.15)
*P*-trend^†^	0.114	0.102
Hyperglycemia		
T1^**§**^ (Reference)	1.0	1.0
T2	0.33 (0.33, 1.75)	0.74 (0.31, 1.76)
T3	0.31 (0.31, 1.66)	0.71 (0.30, 1.69)
*P*-trend^†^	0.440	0.441
Hypertension		
T1^**§**^ (Reference)	1.0	1.0
T2	0.28 (0.45, 1.62)	0.65 (0.26, 1.61)
T3	0.29 (0.54, 1.67)	0.61 (0.24, 1.52)
*P*-trend^†^	0.408	0.288
High hs-CRP		
T1^**§**^ (Reference)	1.0	1.0
T2	0.51 (0.18, 1.44)	0.49 (0.17, 1.43)
T3	0.23 (0.08, 0.61)	0.23 (0.08, 0.62)
*P*-trend^†^	0.002	0.004

*DPI* dietary phytochemical index, *LDL-C* low density lipoprotein cholesterol, *HDL-C* high density lipoprotein cholesterol, *hs-CRP* high-sensitive C-reactive protein, *OR* odds ratio, *CI* confidence interval.

^†^*P* < 0.05 was considered significant.

^**‡**^Adjusted for age, sex, BMI, smoking, and physical activity level.

^**§**^Tertile cut-points of DPI are as follows: first tertile, < 27.3; second tertile, 27.3 to 33.9; third tertile, > 33.9.

## Discussion

Previous studies reported the risk of various chronic diseases and their inverse relationship with the DPI^13^; however, fewer studies evaluated this relationship in the obese population. This research is one of the first studies that investigated the relationship between DPI and oxidative stress markers and cardiovascular risk factors in the obese population. The results of the present study indicated a positive correlation between DPI and consuming olives, olive oils, grains, fruits, and vegetables. DPI was inversely associated with serum concentrations of TG, MDA, erythrocyte SOD activity, and hs-CRP levels. In addition, TAC was positively associated with DPI score. However, no significant relationship was found between the DPI score and FBS, TC, LDL-C, HDL-C, TOS, GPx, CAT, anthropometric parameters, as well as systolic and diastolic blood pressure.

DPI has been linked to the risk of some chronic diseases such as obesity, type 2 diabetes mellitus, metabolic syndrome, cancers, and cardiovascular and inflammatory diseases^13,25–28^. However, the exact mechanisms by which dietary phytochemicals protect against chronic diseases is not entirely understood. The potential beneficial effects of dietary phytochemicals seems to be mediated through down-regulating the inflammatory cytokines, as well as reducing oxidative stress because of their known anti-oxidative and anti-inflammatory properties^29^.

Numerous phytochemicals have been recognized as inducers of antioxidant enzymes in various studies^30^. Hermana et al. in a cross-sectional study on 246 healthy adults found a positive relationship between consumption of fruit and vegetables with TAC and GPx activity in plasma^31^. Another study on 205 prediabetes people also found a positive relationship between dietary intake of fruits and vegetables and TAC and SOD activity^32^. In the present study, DPI score was positively associated with serum TAC levels, but, inversely correlated with SOD activity. Since, SOD enzyme is part of the first line of defense against free radicals, it is expected that this inverse correlation may indicate the compensatory response to decreased oxidative stress by increasing phytochemical intakes through DPI tertiles. These findings support a role for dietary phytochemicals in the antioxidant defense system.

Higher amount of lycopene consumption, a kind of phytochemical, was associated with lower hs-CRP levels^33^. Kim et al. reported an inverse relationship between DPI scores and the likelihood of increasing hs-CRP levels in their study of 18,699 over-19 years Koreans from 2015 to 2018^34^. Results from other studies match these current findings^33,35,36^. However, some other studies found no relationship between DPI and antioxidant or inflammatory biomarkers^37–40^. These contradictions could be due to the different biomarker evaluation methods, compound bioavailability, and the influence of other nutrients on the compound biological effects^41,42^.

Antioxidant properties of dietary phytochemicals have been determined by their capacity to scavenge and interact with reactive oxygen species (ROS) and inhibit enzymes involved in ROS generation^43^. It has been also found that dietary phytochemicals exert their antioxidant effects by activating specific genes involved in encoding antioxidant proteins through the key transcription factor, nuclear factor (erythroid-derived 2)-like 2 (Nrf2), which is known as an important regulator of the antioxidant response^44^.

The anti-inflammatory effects of phytochemicals is mediated through the regulation of various inflammatory cytokines such as interleukins and tumor necrosis factor alpha-α (TNF-α) as well as prostaglandin E2 as a non-cytokine mediator.

Current results indicate that phytochemicals have important antioxidant and anti-inflammatory effects, which reduce oxidative stress and inflammation. Regarding other cardiovascular risk factors including hypercholesterolemia, high LDL-C, low HDL-C, hypertriglyceridemia, hyperglycemia, and hypertension which investigated in the present study, higher scores of DPI was only associated with the lower risk of hypertriglyceridemia. Similar to this finding, in a cohort study on adult population, higher DPI was associated with a lower prevalence of hypertriglyceridemia^45^. Besides, a meta-analysis of 49 studies showed that plant-based diets are associated with improved plasma lipids^46^. Kim and Park also reported a significant association between higher DPI and a lower prevalence of hypertriglyceridemia, hyperglycemia, and high blood pressure^25^. Some evidence is also available regarding higher DPI and lower FBS levels in healthy people^47,48^. However, in one study on Iranian adults, no relation between DPI and serum lipid profile was found^49^. Besides, a cross-sectional study of 850 adults with metabolic syndrome reported no relationship between following a phytochemical-rich diet and the metabolic syndrome components^49^. Furthermore, few other cross-sectional studies found no significant relationship between higher intakes of flavonoids, polyphenols, and carotenoids and the metabolic syndrome components^50–52^.

These contradictions in the current evidence are probably because of heterogeneity in study designs, measured outcomes, sample sizes, specific food culture and habits, study population socio-demographic characteristics, and used assessment tools. Therefore, there were challenges in generalizing these results to other societies.

Population-based (obese people) design, data analysis after potential confounder modification, using valid questionnaires, and in-person interviews by trained nutritionists are the major strengths of this study. However, this study has some limitations, such as the impossibility of determining causality due to the cross-sectional nature of this study, the lack of native food databases for plant nutrients, and inherent DPI limitations, such as not considering some foods with high phytochemical values that have no energy (e.g., spices, green and black tea) and ignoring the phytochemical compound type and quality.

## Conclusion

This study confirmed that there was a significant inverse association between DPI and oxidative stress, inflammation, and hypertriglyceridemia as CVD risk factors in obese population. Further randomized clinical trials and large-scale prospective cohorts are needed to validate these current findings using dietary data with low measurement errors.

## Data Availability

The data that support the findings of this study are available from the corresponding author upon reasonable request.
